# Medicare coverage choice is not neutral: how policy design shapes beneficiary enrollment

**DOI:** 10.1093/haschl/qxag113

**Published:** 2026-05-14

**Authors:** Angela Liu, Isabelle Jouve

**Affiliations:** Department of Health Policy and Management, Johns Hopkins Bloomberg School of Public Health, Baltimore, MD 21205, United States; Johns Hopkins University, Baltimore, MD 21218, United States

**Keywords:** Medicare, enrollment choice, affordability, benefits

## Abstract

Medicare enrollment has shifted substantially toward Medicare Advantage (MA), rising from 33% in 2016 to over half of beneficiaries by 2025. While often interpreted as reflecting beneficiary preference, Medicare coverage choice is not neutral; policy design systematically advantages MA at the point of enrollment. Medicare's choice architecture concentrates salient financial protections and supplemental benefits within MA while deferring the consequences of managed care restrictions as future considerations, when switching options are limited. We identify 3 domains that create structural asymmetries between MA and Traditional Medicare (TM): affordability and benefit offerings, integrated plan design, and information and marketing. Together, these features make MA's advantages more visible at enrollment, through out-of-pocket caps, supplemental benefits, streamlined coverage, and third-party steering, while TM remains comparatively fragmented. We outline policy options to address these imbalances, including introducing an out-of-pocket cap in TM, expanding benefits, simplifying coverage design, improving decision and support tools, and strengthening oversight of marketing and brokerage practices. Leveling the playing field is not about steering beneficiaries toward TM or MA, but about ensuring that Medicare's growing reliance on enrollee choice rests on a foundation of comparability, transparency, and informed decision-making.

Key points

- Medicare Advantage (MA) enrollment is often interpreted as beneficiary preference, yet policy design shapes Medicare enrollment choice.
- MA's advantages are more visible at the point of enrollment, through out-of-pocket caps, supplemental benefits, streamlined coverage, and third-party steering. Over time, these dynamics can lock beneficiaries into coverage pathways that are difficult to reverse and make policy debates about program performance harder to interpret.
- Leveling the playing field is therefore not about steering beneficiaries toward one program or another, but about ensuring that Medicare's growing reliance on consumer choice rests on a foundation of comparability, transparency, and informed decision-making.

## Introduction

Over the last decade, Medicare enrollment has shifted significantly from Traditional Medicare (TM) to Medicare Advantage (MA): 33% of beneficiaries enrolled in MA in 2016 compared to 54% in 2025.^1^ While this trend may suggest that enrollees prefer MA, MA concentrates visible financial protections and supplemental benefits at the moment of choice, while deferring the consequences of managed care restrictions, such as prior authorization policies and narrow provider networks. Medicare coverage choice is not neutral—policy design systematically advantages MA enrollment.

This is not to suggest that MA is not the appropriate choice for some beneficiaries. Rather, the concern is that beneficiary decision-making may reflect preferences for visible benefits, such as decreased premiums, shaped by an uneven choice environment that favors MA, more than clinical needs. When salient benefits are restricted to one program, plan choice is complex, and marketing and broker incentives are misaligned with beneficiary interest, enrollment patterns may reflect the structure of the marketplace rather than informed consumer choice.

Leveling the playing field between TM and MA choice matters because Medicare coverage decisions carry long-term consequences for beneficiaries’ access to providers, financial risk, and care experiences, while also shaping federal spending and the competitive dynamics of the Medicare program.

This paper identifies 3 policy domains that skew Medicare's choice architecture toward MA enrollment: (1) affordability and benefit offerings asymmetry, (2) integrated plan design asymmetry, and (3) information and marketing asymmetry. For each domain, we discuss policy options and key considerations (Table 1).

**Table 1.** Medicare coverage choice asymmetries, drivers, policy recommendations, and potential concerns

Asymmetry	Drivers of asymmetry	Recommendation	Description	Potential concerns
Affordability and benefits offering	Narrow Medigap enrollment windows and individual underwriting	Reform Medigap at the state-level	Establish requirements for Medigap community rating or guaranteed issue at the state-level	Concerns include higher Medigap premiums and Medigap market instability, and increased Medicare spending
	Payment policies that help fund the OOP cap in MA	Establish an OOP cap in TM	Institute an OOP cap in TM, with considerations for how it interacts with enrollee income, as well as Medigap and Medicare Advantage markets	Concerns include increased Medicare spending and impacts on the federal budget
	Payment policies that help fund MA supplemental benefits	Add supplemental benefits to TM	Offer supplemental benefits in TM comparable to those in MA, including hearing, vision, dental, and non-medical benefits	Concerns include increased Medicare spending and TM premiums
	Widely varying MA supplemental benefit packages and cost-sharing levels	Standardize MA benefit packages	Create standardized options for Parts A and B cost-sharing and supplemental dental, vision, and hearing benefits for MA plans	Concerns include disruption in benefit offerings during initial implementation
Integrated plan design	Statutory requirement for MA coverage to integrate Parts A, B, and D	Streamline and integrate TM coverage	Bundle TM coverage so beneficiaries enroll in Parts A, B, and D under one plan	Concerns include limited Part D plan options
	Narrow Medicare enrollment windows and unclear eligibility notifications	Simplify enrollment processes and improve information about eligibility	Simplify enrollment periods and implement clearer eligibility notifications	
Information and marketing	Limited government tools to support enrollee decision-making	Improve the Compare Coverage Options tool	Add OOP spending estimates and Medigap information to Compare Coverage Options tool	
	Regulatory framework allowing broker incentives for MA enrollment and advertising that steers beneficiaries toward MA	Strengthen oversight of third-party marketing and brokerage	Impose greater restrictions on misleading marketing and require brokers to act in beneficiaries’ financial interests	
		Expand resources for unbiased enrollment guidance	Increase funding and public awareness for the State Health Insurance Assistance Program and the Senior Medicare Patrol	Concerns include increased Medicare spending and impacts on the federal budget

### Affordability and benefits offering asymmetry

Many of MA's additional financial protections and benefit offerings are supported by payment policies that have resulted in higher MA payments relative to TM. These payment policies include benchmarking against TM, risk-adjusting based on reported diagnoses, and payment bonuses that may not accurately reflect care management. While appropriate payment amounts continue to be widely debated, we spotlight asymmetries that would persist even if payment reform was achieved.

#### Affordability asymmetry

MA plans offer lower premiums and more salient financial protections compared to TM. One policy decision was to include a federally mandated minimal out-of-pocket (OOP) cap in MA. TM beneficiaries face uncapped cost-sharing, including 20% coinsurance for outpatient services.^2^ TM beneficiaries can purchase supplemental insurance through Medigap plans, which cover varying levels of cost-sharing. However, most beneficiaries have only a 6-month window to enroll without individual underwriting. Many are not aware of this window, and for those who are aware, purchasing Medigap requires anticipating future healthcare needs and financial risk.^3^

Because MA guarantees an OOP cap while TM relies on optional supplemental coverage,^2^ financial risk protection is automatically guaranteed in MA but fragmented and optional in TM—a structural affordability asymmetry that shapes beneficiary enrollment.

Two policy approaches could address this asymmetry: First, states can reform Medigap policies to expand access to Medigap coverage. In fact, several states have recently passed Medigap community rating or guaranteed issue requirements, though these policies may increase Medigap premiums and raise concerns about market stability.^4^

Second, policymakers could implement an OOP cap in TM.^5^ Key considerations include the cap level and how it interacts with enrollee income. Policymakers must also consider how a TM OOP cap would impact the MA, Medigap, and Part D markets. While such a cap would make TM more attractive and reduce reliance on Medigap for financial protection, it would also increase Medicare spending and impact the federal budget.

#### Benefits offering asymmetry

MA plans offer benefits not included in TM, such as hearing, vision, and dental coverage,^2^ which are arguably essential for the Medicare population of older adults. Many MA plans also include non-medical supplemental benefits, such as gym memberships and transportation services.^2^ These additional benefits can make MA more attractive at the point of enrollment—a coverage asymmetry that tilts the playing field toward MA.

One policy response is to add supplemental benefits to TM comparable to those offered in MA. Since these additional benefits could result in greater Medicare spending and premiums, important policy considerations include the benefit allowance amounts, specific services that would be covered, and how these additional benefits would interact with enrollee premiums and cost-sharing.

Even within MA, benefit offerings vary widely across plans. Standardized MA benefit packages could improve comparability and help beneficiaries make more informed decisions.^6^ Additionally, MA insurers are now required to report utilization rates for supplemental benefits so beneficiaries can better evaluate their value when choosing coverage.

### Integrated plan design asymmetry

MA insurers offer integrated plans, bundling Part A, Part B, and prescription drug coverage under one plan. In contrast, TM beneficiaries enroll in Part A and Part B separately, then choose among many Part D plans for prescription drug coverage.^2^ This fragmentation can make TM more difficult to navigate, and MA's plan integration and simplicity in choice further tilts the playing field toward MA.

Simplifying TM coverage could reduce this asymmetry. A more streamlined design—including integrating Part A, Part B, and Part D—could guarantee simple and comprehensive coverage. Including OOP protections would further simplify choices by reducing reliance on Medigap. Additional policy levers include clearer eligibility notifications, simplified enrollment periods, and auto-enrollment strategies designed to help beneficiaries select appropriate coverage.^6^

### Information and marketing asymmetry

Because choosing Medicare coverage is complicated, beneficiaries may rely on external information sources. Government tools, such as the Compare Coverage Options website or the MA Star Ratings program, aim to support decision-making but have important limitations. For example, Compare Coverage Options website does not provide an OOP estimate in TM, with or without Medigap coverage, while the MA Star Ratings program has been criticized for failing to effectively signal high-value care.^7^

Improving government decision tools could help this asymmetry. Compare Coverage Options should include estimates of OOP spending, including deductible amounts, for beneficiaries with chronic health conditions and better information about Medigap and underwriting restrictions.

When government tools fall short, beneficiaries often rely on brokers or are especially susceptible to marketing strategies. In both cases, the playing field is tilted toward MA. During the 2023 annual Medicare open enrollment period, Medicare-related ads aired more than 643 000 times, and 85% of those were for MA plans; most of the remaining ads promoted Medigap.^8^ Third-party intermediaries have been criticized for steering beneficiaries to MA plans, potentially reflecting financial incentives rather than beneficiary preferences or care needs.^9^

CMS should strengthen oversight of third-party marketing and brokerage practices. Policies could include restrictions on misleading marketing and requirements that brokers act in beneficiaries’ best financial interests. Additionally, expanding funding and public awareness for the State Health Insurance Assistance Program and the Senior Medicare Patrol would further support unbiased enrollment guidance.^9^

## Conclusion

Payment reform alone will not resolve the deeper asymmetries embedded in how beneficiaries experience enrollment choice.^10^ Without addressing these asymmetries, Medicare risks evolving into a system where enrollment trends are driven more by how options are designed and marketed rather than by their underlying value. Over time, this dynamic can lock beneficiaries into coverage pathways that are difficult to reverse and make policy debates about program performance harder to interpret. Leveling the playing field is therefore not about steering beneficiaries toward TM or MA, but about ensuring that Medicare's growing reliance on consumer choice rests on a foundation of comparability, transparency, and informed decision-making.

## Supplementary Material

qxag113_Supplementary_Data
